# Understanding Employee Voice Behavior Through the Use of Digital Voice Channel in Long-Term Care: Protocol for an Embedded Multiple-Case Study

**DOI:** 10.2196/48601

**Published:** 2024-02-02

**Authors:** Anja Kepplinger, Alexander Braun, André Fringer, Martina Roes

**Affiliations:** 1 Department of Nursing Science, Faculty of Health Witten/Herdecke University Witten Germany; 2 Institute Nursing Science, Department of Health Sciences IMC University of Applied Sciences Krems Krems Austria; 3 Institute Health Management IMC University of Applied Sciences Krems Krems Austria; 4 Institute for Management and Economics in Healthcare UMIT Tyrole Hall Austria; 5 Institute of Nursing, School of Health Sciences, Zurich University of Applied Sciences ZHAW Winterthur Switzerland; 6 Deutsches Zentrum für Neurodegenerative Erkrankungen (DZNE) Witten Germany

**Keywords:** digital voice channel, employee participation, employee voice, health care provider, home care facilities, long-term care, nursing home

## Abstract

**Background:**

Specific challenges in the health care sector, such as hierarchical structures, shortages of nursing staff, and high turnover of nursing staff, can be addressed by a change process of organizational culture into shared governance. Data from business organizations show that the use of digital voice channels provides employee voice. This approach makes concrete the opportunity for employees to raise their voices by answering surveys and making comments in an anonymous forum, which subsequently positively influences staff turnover and sick leave. Since there is no clear understanding of how a digital voice channel can be used in long-term care to address employee voice, a research gap has been identified.

**Objective:**

The purpose of ADVICE (Understanding Employee Voice Behavior; the acronym for this study) is to understand how the use of a digital voice channel performs in long-term care (residential long-term care and home care facilities). The aim of this study is to understand how the digital voice channel can support staff in making their voices heard and to see what managers need to use the voice channel to change the work environment.

**Methods:**

An embedded multiple-case study will be used to explore the experiences of 2 health care providers who have already implemented a digital voice channel. ADVICE is organized into two main phases: (1) a scoping review and (2) an embedded multiple-case study. For this purpose, focus group interviews with employees, discursive-dialogical interviews with managers, meeting protocols, and data from the digital voice channel will be analyzed. First, all units of analysis from every embedded unit will be separately analyzed and then comprehensively analyzed to obtain a case vignette from every embedded unit (within-analysis). In the second stage, the analyzed data from the embedded units will be compared with each other in a comparative analysis (cross-analysis).

**Results:**

The results will provide insight into how digital voice channels can be used in long-term care to address employee voice. We expect to find how the digital voice channel can empower nurses to speak up and, consequently, create a better work environment. Data collection began in August 2023, and from a current perspective, the first results are expected in summer 2024.

**Conclusions:**

In summary, the results may help to better understand the use of a digital voice channel in the health care sector and its transformative potential for leadership. At the organizational level, research can help to improve the attractiveness of the workplace by understanding how to give employees a voice.

**International Registered Report Identifier (IRRID):**

PRR1-10.2196/48601

## Introduction

### Overview

The International Council of Nurses points out that there is a need to reduce the dropout rate in nursing and promote the loyalty of employees to their health care provider [1]. Data from the Austrian Work Climate Index that are related to working conditions in the nursing professions describe that 65% of nurses intend to leave their current position, and 15% have plans to change their profession [2]. Furthermore, according to the NORDCARE report on working conditions in long-term care in Austria [3], the working conditions perceived by nurses have declined in recent years. As justifications, the respondents noted the increase in work demands, staff shortages, and lack of time for caring activities. A low level of autonomy, especially among nurses, is highlighted by the collected data. Nursing assistants and home assistants state that they feel left alone with the responsibility for people in need of care. The NORDCARE report [3] findings are corroborated by data from the Austrian Work Climate Index [2], indicating that 44% of employees lack or have inadequate chances to participate in work activities, which may negatively impact on-job satisfaction.

### Background

To promote high-quality, person-centered care in the context of challenges related to nursing turnover as well as the different needs and requirements of different generations, health care providers need to invest in creating a productive work environment [4]. To sustainably change the culture in an organization, the interplay of transformational leadership, shared governance, and action processes is needed [5]. In particular, transformational leadership is presented as a leadership style that is action-oriented and enables development and change through shared governance. Shared governance is seen as a management approach in the sense of enabling all stakeholders and employees to have a voice in decision-making processes [5,6].

Recommendations to implement a change process in organizations using transformational leadership and shared governance are supported by the positive impact of the Pathway to Excellence in Long-Term Care (PTE-LTC) program. This is a special Magnet Recognition Program of the American Nurses Credentialing Center for long-term care. The PTE-LTC program is based on 6 pathway standards: professional development, shared decision-making, leadership, well-being, quality, and safety. The goal is to empower and give frontline staff a voice, promote staff participation in improving outcomes, create a sustained culture, strengthen interprofessional collaboration, and ensure the well-being of employees [7]. Key differences between the Magnet Recognition Program and the PTE-LTC program are that the PTE-LTC program includes a person-centered model of care and unlicensed assistive personnel such as nurse assistants [7]. Research indicates that the focus in health care organizations on a better work environment and higher engagement in shared governance decreases rehospitalization and lowers nurse burnout and job dissatisfaction levels [8-10]. A systematic review shows that certified magnet hospitals, compared with nonmagnet hospitals, have higher job satisfaction levels, less burnout, lower turnover, lower rates of nurse shortages, higher quality of care, and better patient outcomes [11].

The “Relationship-Centered Team Nursing Model for Care Delivery in Nursing Homes” is a nursing care delivery model that attempts to address workforce challenges in long-term care. The model describes 4 assumptions. The first is to skillfully manage a multilevel team with a focus on care coordination and communication; the second is to empower and develop staff and delegate appropriately and responsibly; the third is the requisite preparation of nursing staff to deliver person-centered care; and the fourth is nursing leadership. In conclusion, the model addresses an evidence-based approach, clear communication, and staff empowerment [4]. Clear communication means, according to Siegel et al [4], open, clear, and timely communication between staff, residents, their families, and the involved structures.

The PTE-LTC program and the “Relationship-Centered Team Nursing Model for Care Delivery in Nursing Homes” emphasize the importance of empowering frontline staff so that in practice they can have a participatory voice that aims to influence decisions, communicate ideas, communicate residents’ needs and requirements, and initiate change [4,7]. This participatory element of workplace democracy is known as employee voice. Employee voice refers to all organizational structures, mechanisms, or practices in which employees participate and try to influence their work and the performance of their organization [12,13]. The term employee voice has a long tradition in organizational research and is variously defined in the literature [14]. A summary by Dundon et al [15] describes the concept of employee voice in terms of four characteristics: (1) addressing dissatisfaction, opinions, or suggestions; (2) expressing a collective organizational culture; (3) managing decision-making processes; and (4) demonstrating a cooperative relationship between management and employees in an organization [15]. Elizabeth Wolfe Morrison, a professor of management and organization at the Stern School of Business, New York University, defines employee voice as “informal and discretionary communication of ideas, suggestions, concerns, problems, or opinions about work-related issues, with the intent to bring about improvement or change” [14].

The literature on employee voice addresses promotive and prohibitive factors of voice, such as how supervisors respond to that behavior and the effects that voice has. For example, Holland et al [16] and Kee et al [17] show that mechanisms that address the voice of employees can reduce burnout, promote employee well-being [16], and influence organization development through structures that employees are encouraged to explore and give feedback [17]. However, according to Morrison [14], there is a need to consider how informal employee voice behavior is affected by formal opportunities and channels for voicing, both individually and collectively.

Recent developments in digital and communication technology have opened new opportunities to address employee voice [18-20]. For example, the social and economic scientist and sociologist Ellmer and Reichel [19] describe the possibility of a company mirror or digital voice channel. This digital communications technology enables voice by allowing employees to periodically report their work satisfaction through minisurveys and make anonymous comments in miniforums. Ellmer and Reichel [19] show in their case study that employees are encouraged to speak up and discouraged from an affordance perspective. In this context, the affordance perspective refers to the definitions of Gibson [21] and Norman [22] and means analyzing the possibilities for action that an artifact offers to an actor, as well as the constraints. The possibilities that an artifact can support or hinder a person in achieving a goal. These results indicate that the interplay of material and social aspects, that is, the characteristics of the voice channel and the responses of the management embedded in the respective organizational context, lead to employees perceiving the channel as either promoting or restricting speech. This further leads to whether employees feel encouraged or discouraged to speak up or not speak up [19]. In summary, a digital voice channel can have a positive impact on the identification and attachment of the employees with the company, leading to a reduction in turnover and sick leave, which depends on job satisfaction [16], perception of autonomy, the influence of organizational context [23], and the perception of safety [24] and dependencies [19]. These results indicate that a digital voice channel is not sufficient on its own; it must be seen as a part of an overall approach to shared governance [19].

Based on these findings, due to the increasing challenges related to nursing turnover and the different needs and requirements of different generations, new measures are needed to meet the increasing demand for nurses to ensure high-quality, person-centered care.

To date, the use of a digital voice channel in the health care sector has received little attention. Improvements in the health care structure, IT, organization, and legislation are needed to ensure that health care is error-free, efficient, and effective. The Health & Care Expert Council recommends investing in the promotion of digital health literacy, that is, empowering people to deal competently with the digitalization of health care [25]. Available data indicate that in the health care sector, it can be difficult for employees to have a voice due to hierarchical structures [17,26]. For example, Kee et al [17] research how nursing assistants can develop voice behavior that transcends hierarchical levels. The study results show that by training in personal reflection as well as individual coaching, nursing assistants are able to influence organizational structures and initiate change. As a result, it is necessary to understand the wider context of social processes in an organization to address the voice of employees. For example, to know that psychological safety and commitment-based safety management have an impact on whether nurses are willing to raise concerns and be aware that psychological safety is a social-cognitive variable that varies between individuals within the same work context [27-29]. Research also exists regarding the content and structure of simulation training on speaking up in health care organizations [30,31]. It should be noted that the evidence on simulation training has mainly focused on inpatient and emergency settings, operating theaters, and intensive care units and not on long-term care. Martin et al [32] suggest that relying solely on formal channels may discourage some individuals from speaking up, hence the importance of complementing with informal methods. It is recommended to use a combination of both to encourage a greater variety of voices to be heard. Further studies have examined the implementation of a new role called “guardian” to promote voice in the organization. The research indicates that this role should not only focus on formal acts of voice such as support and advice. Instead, a relationship role between the guardian and colleagues is needed, as well as a well-defined role between the existing channels for voice [33]. As Jones et al [34] and O’Donovan and McAuliffe [35] noted, it is unlikely to find a 1-size-fits-all approach to creating a culture where health care professionals can raise their voice. In summary, it must be mentioned that in the health sector, the term employee voice is not used consistently. The existing literature on the use of voice in health care focuses on the use of voice in the context of patient safety and error reporting. In summary, the current review by Lainidi et al [36] points to the lack of a comprehensive theory of employee well-being and communication in the health care sector, which leads to heterogeneous mechanisms and an unclear understanding of the phenomenon of employee voice in health care. Lainidi et al [36] argue that understanding the concepts of voice and silence from both organizational and employee perspectives is crucial, as is the need for different solutions for different contexts. This means that individual approaches and evaluations are needed to gain a deeper understanding of employee voice, especially in the long-term care sector.

In their systematic review, Mair et al [37] note that the published literature focuses on organizational aspects yet neglects the broader social framework that needs to be considered when introducing new technologies. More recently, Krick et al [38] have recommended more evaluations of digital technologies in a real-life setting. It is not about what is technically possible; rather, it is about how new digital technologies in the daily routine can be designed to consider staff needs and preferences [20,39]. Ong et al [40] address this research gap and analyze the implementation of a digital patient feedback system using normalization process theory (NPT) from May and Finch [41] and May et al [42]. NPT is a middle-range theory that provides a sociological framework to understand social processes. The theory was developed to understand how a practice becomes or does not become routinely embedded in social contexts. A total of 4 key components of the theory are of relevance: coherence, cognitive participation, collective action, and reflexive monitoring. These 4 components define the process of normalization. Coherence refers to the fact that the practice is an ensemble of beliefs, behaviors, and acts that are defined by a set of ideas about its meaning, uses, utility, and socially defined and organized competencies. The nature of embedding depends on the work that defines and organizes a practice as a cognitive and behavioral ensemble and requires that actors collectively invest meaning in it. Cognitive participation means that normalization depends on the work that defines and organizes the actors involved in a practice. Collective action includes the chain of interactions, that is, a place of mental and material work that deals with organizing and enacting a practice. Reflexive monitoring means the continuous formal and informal evaluation of the patterns of collective action and their outcomes by all participants [41,42]. Collectively, NPT provides an appropriate conceptual framework to understand issues relating to routinization of new technology [37,43]; it will therefore be used in the ADVICE (Understanding Employee Voice Behavior; the acronym for this study) study as the theoretical lens.

This study will fill a gap in knowledge because it aims to understand how staff and managers use a digital voice channel in the social context of health care providers who offer long-term care services, to subsequently understand how employee voice behavior is affected by the digital voice channel, and to explore what opportunities arise for the employees and for the organization through a digital voice channel. The digital voice channel is a browser-based software-as-a-service system that organizations can purchase on a subscription basis. The founders of the digital channel provider aim to empower employees to actively participate in the improvement and decision-making processes in their workplace by giving them the opportunity to (1) periodically report on their job satisfaction through minisurveys and (2) post anonymous comments in a miniforum. On the user interface, a dashboard with a graph shows the progression of a sentiment metric over time, allowing analysis of current and past sentiment levels in the organization. The sentiment metric is based on responses to anonymous minisurveys covering 6 categories: well-being, health, collaboration, potential development, work activity, and organizational culture. The standard pool of questions can be supplemented by various additional pools. Employees can select from a Likert scale ranging from 1 to 7 (1=does not apply at all to 7=completely true). To gather votes, a link to minisurveys (ranging from 3 to 10 questions) is sent to all employees through email at specified intervals (weekly or monthly), depending on the organization’s preference. Managers are alerted when new votes or anonymous comments are available. This information, combined with the dashboard, can be used to identify and act on trends and dynamics in the team early on, allowing decisions to be made based on this information.

### Aim of This Study

The aims of the study are to investigate the phenomenon of the digital voice channel in a real-life context and to explore opportunities and capture experiences to enable a systematic and professional approach in the future. This means that it is about generating new knowledge to make the new techniques usable and normalized in long-term care. The overall question is as follows: How does the use of a digital voice channel support opportunities to affect employee voice behavior in long-term care?

For the planned scoping review, the following question is relevant: Which opportunities are mentioned in the literature to address employee voice in health care providers?

For the embedded multiple-case study, further questions are relevant:

What are the experiences in long-term care with the use of a digital voice channel? How is the normalization process described in long-term care, and how do they differ?

## Methods

### Overview

To answer the research questions, an embedded multiple-case study will be chosen. Case studies can be used to analyze a phenomenon from multiple perspectives in a real-life context [44] to gain a deeper understanding [45]. The case study approach is especially appropriate when there is little understanding of a phenomenon in a certain context [44]. According to Yin [44], case study research is described by 5 components: questions, propositions, cases, data collection, and data analysis. Propositions in ADVICE will result from the scoping review and from NPT. The contexts are 2 health care providers that have already implemented a digital voice channel. These 2 long-term care providers describe the context in which several residential long-term care and home care facilities (cases) operate. Both providers use the same digital voice channel.

To analyze the social processes related to the use of a digital voice channel in health care providers, multiple data sources are necessary to create dense descriptions [44,46]. For this purpose, focus group interviews with employees, discursive-dialogical interviews [47] with managers (plus field notes), meeting protocols, and data from the digital voice channel will be analyzed (units of analysis). The qualitative data and comments in the survey will be analyzed by a deductive-inductive content analysis approach developed by Schreier [48]. Data from the digital voice channel include response rates and frequency of the mentioned topics. These quantitative data are displayed in the digital voice channel and provided by the health care provider. In summary, a phenomenon (digital voice channel) will be analyzed in a real context (2 health care providers) to achieve a deeper understanding of how a digital voice channel becomes a daily routine, that is, normalized [49]. It should be noted that the case definition or units of analysis, as well as other aspects of the research design in a case study, may be revised during data collection [50].

**Figure 1:**
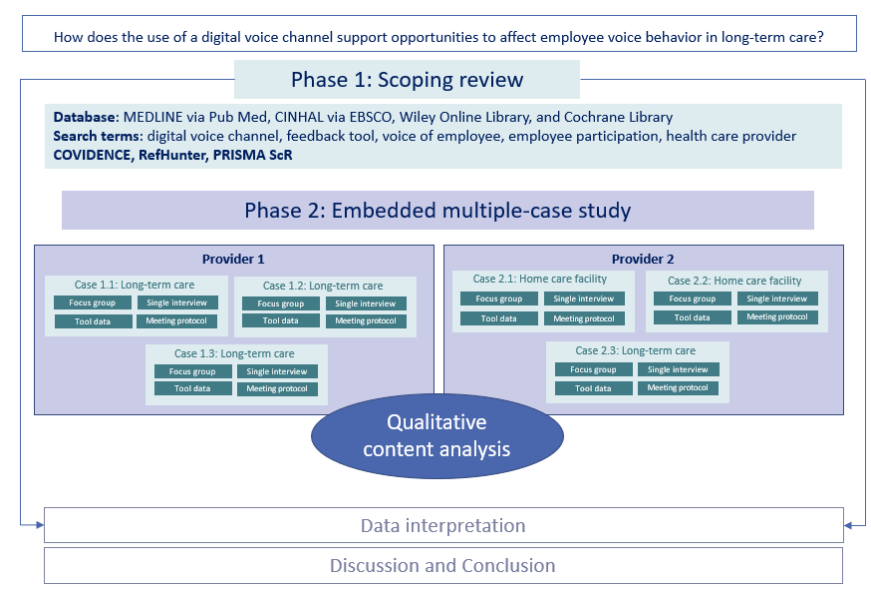
Overview of the research phases of the ADVICE (Understanding Employee Voice Behavior) study.

ADVICE is organized into two main phases: (1) the completion of a scoping review and (2) the conduction of an embedded multiple-case study, as shown in Figure 1.

### Phase 1: Scoping Review

The scoping review [51] aims to provide an overview of the literature on employee voice in health care providers and the use of opportunities or interventions such as voice channels, assessments, tools, feedback instruments, feedback training, or meetings to promote having a voice in the organization. To develop the search components, the question is integrated into the PICO (population, interest, and context) schema for qualitative studies [52], as shown in Textbox 1. The scoping review will be conducted using the PRISMA ScR (Preferred Reporting Items for Systematic Reviews and Meta-Analyses extension for Scoping Reviews) [53].

**PICO schema.**

**P: population**

- Health care provider

**I: interest**

- Opportunities like digital voice channel

**Co: context**

- Employee voice

The search process took place in the period ranging from September 2022 to April 2023 in the MEDLINE through PubMed, CINHAL through EBSCO, Embase, Wiley Online Library, and Cochrane Library databases. Furthermore, the reference lists of relevant results will be screened and identified through Google Scholar. The following search terms will be used for the search: digital voice channel, feedback tool, company mirror, feedback, assessment, tool, voice of employee, employee participation, health care provider, long-term care, and home care facilities. Furthermore, the search terms and their synonyms will be linked to search strings using Boolean operators dependent on the databases, as well as MeSH (Medical Subject Headings) terms. After a sensitive search, the research team will consult with each other and adapt the search string. To support the search, the researchers will use the manual for literature search in specialized databases, namely, RefHunter [54]. The inclusion and exclusion criteria shown in Textbox 2 have been derived from the objective and research questions. There will be no restrictions regarding the currency of the literature included.

**Inclusion and exclusion criteria for scoping review.**

**Inclusion criteria**

- Health care provider, residential long-term care, and home care facilities
- Digital voice channel, digital feedback tools, feedback tools, evaluation, assessment, and tools
- Employee voice, employee participation, and team learning
- Qualitative and quantitative studies (all scientific studies)
- English and German

**Exclusion criteria**

- Other settings similar to business areas
- Summaries and discussions
- Other languages

Covidence is a software for managing and simplifying reviews. The program Covidence supports the review process after the removal of duplicates and enables the screening of the literature by 2 reviewers independently [55]. Specifically, the titles and abstracts will be reviewed against the inclusion and exclusion criteria to identify appropriate studies. In the next step, the full texts of the relevant studies will also be reviewed by 2 reviewers independently. Any disagreements about the eligibility of a study will be resolved by discussion in the research group. For data extraction, a standardized data extraction form will be developed. The form will be tested with the first 3 results and discussed in the research group. The items included in the description of each article will be selected following the Template for Intervention Description and Replication (TIDieR) [56] and Criteria for Reporting and Evaluation of Complex Interventions (CReDECI 2) in health care [57]. Based on the iterative character of the scoping review, all changes will be documented in the protocol [51]. The results of the scoping review will be used to develop propositions and the interview guide used in phase 2 of the ADVICE study.

### Phase 2: Embedded Multiple-Case Study

#### Overview

Based on the results of the scoping review, this research phase aims to go into the field and immerse itself in the day-to-day work of the cases to understand the social processes that involve the promotion of employee voice. A detailed description of the components of Yin’s [44] case study research design follows.

#### Casing

As shown in Figure 1, the ADVICE sample includes 6 different cases that are situated in 2 different contexts (providers 1 and 2). After Sandelowski [50], the cases for a case study are made, not found, through an iterative and theoretical process called casing. The boundary of the cases can be determined by the phenomenon of the use of the digital voice channel and the same scope of long-term care (content boundary). In these cases, the temporal boundary refers to the time of digital voice channel implementation. Starting with the time of implementation in provider 1, (2020) and provider 2 (2021), the end time describes the end of the data collection from the current point of view (2024) [44].

Provider 1 is a health care provider with approximately 1000 employees in different residential long-term care facilities in Austria. A digital voice channel has been used there since 2020. From the current perspective, this health care provider is defined by 3 cases through 3 long-term care facilities.

Provider 2 is a health care provider of several residential long-term care and home care facilities with approximately 5000 employees in Austria. The health care provider has been using a digital voice channel in a pilot phase in a few facilities since 2021. From the recent perspective, this health care provider is defined by 3 cases, namely, 1 residential long-term care and 2 home care facilities. In this case, a long-term residential care facility is an institution where people who need care can live or stay for a period of time under the responsibility of professional health care workers. Home care facilities provide professional care to patients in their own homes. The research team is aware of the different settings (residential long-term care and home care facilities) in the embedded units, which represents a limitation. The use of the different samplings can be argued by the fact that the focus of the analysis is the use of a digital voice channel, as well as employee voice behavior, in long-term care providers. Thus, it is not about the setting of health care providers per se; rather, the commonality is the same setting of long-term care, the same digital voice channel, and therefore, the same organizational culture with different characteristics. Additionally, based on the different settings, additional data, such as individual characteristics of the specific areas and differences, can be generated. The concrete number of employees and number of beds in the embedded units cannot yet be described, as the recruitment phase is only just beginning.

#### Data Collection

##### Overview

Based on the recommendation of Yin [44], data collection will take place using multiple sources of evidence. Consequently, data will be collected in the form of focus group interviews, discursive-dialogical interviews [47], meeting protocols, and data from the digital voice channel (anonymous survey data and free comments). During the whole research process, field notes and written memos concerning methodological, personal, and case-related issues will be taken by the first author [58]. These different data are, according to Yin [44], the embedded units of analysis.

Specifically, data from the digital voice channel will be searched concerning the key mechanism of reflexive monitoring drawn from NPT [41,42], for example, how benefits or problems with the digital voice channel are identified or measured.

##### The Interviews

The interviews will include managers, nurses, and social care professionals in each case. Based on the Austrian Nursing Practice Act, nurses are defined as registered nurses with a diploma and a bachelor’s degree (full-time training, 3 years), nursing specialist assistants (full-time training, 2 years), and nursing assistants (full-time training, 1 year). In addition to nurses, social care professionals also work in residential long-term care and home care facilities. There are 3 levels of qualification based on the Social Care Profession Act (home assistant, social care workers, and certified social care workers). Home assistant complete 200 hours of theoretical course and 200 hours of practical training. Social care workers complete a 1200-hour theoretical course and 1200 hours of practical training. Certified social care workers have an 1800-hour theoretical course and 1800 hours of practical training.

It should also be noted that medical care in residential long-term care in Austria varies greatly according to the laws of the individual federal states. In most facilities, it is provided by general practitioners. This means that general practitioners are not employed by the health care provider; they provide medical care by visiting their patients. For example, it is possible for nurses in residential long-term care facilities to have different general practitioners to contact because residents bring their own general practitioners with them. This applies to other health care professionals like physiotherapy, occupational therapy, and speech and language therapy. In summary, this indicates that physicians and health care professionals are not employed in the health care providers and therefore are not using the digital voice channel.

In addition, managers in different positions will be interviewed. The inclusion and exclusion criteria used for the interview participants are summarized in Textbox 3.

The interviews will focus on the following key NPT mechanisms*:* coherence*,* cognitive participation*,* and collective action [41]. In the context of ADVICE, coherence means asking about sense-making both as an individual component and as a team component. For example, do the participants see a sense in the use of the digital voice channel or not? Do they see a benefit for themselves and for the team? Cognitive participation includes topics aiming to understand the initiation, promotion, and legitimation of participation. Social norms and formal or informal rules in the team play a role in this key mechanism. For example, are facilitators involved in which role they either have or do not have, and is there a collective commitment in the digital voice channel or not? The collective action mechanism refers to all measures taken to use the digital voice channel. Examples include competencies for workability, such as knowledge work, which is necessary to build trust, and digital competencies [41,59].

**Inclusion and exclusion criteria for interviews.**

**Inclusion criteria**

- Women, men, and diverse
- Nurse (registered nurse, nursing specialist assistant, and nursing assistant)
- Home assistant, social care workers, and certified social care workers
- Manger (nursing director, nursing manager, department manager, deputy manager, head of nursing department, and head of nursing unit or nursing home)
- Employed for at least 6 months in 1 of the 2 health care providers that uses a digital voice channel
- German or English

**Exclusion criteria**

- Employees who do not work in 1 of the 2 health care providers or who are retired nurses or nursing students
- Employed for less than 6 months in 1 of the 2 health care providers that uses a digital voice channel
- Other languages

During the focus group interviews, nurses will have the opportunity to discuss the use of the digital voice channel. The aim here is to obtain a picture of what nurses need to be able to give a voice and the impact of the voice channel on their voice behavior. Focus group interviews will be conducted with nurses who work in one of the cases of provider 1 or 2 and do not have a management position. Approximately 6 focus groups will be conducted, each with at least 5-6 individuals in each group. Heterogeneity will be considered in the recruitment process. With attention to the professional qualification mix, every professional nursing group will be represented. The focus group interviews will be moderated by the first author with the help of an interview guide based on the results of the scoping review and NPT.

The single interviews will be conducted by the first author in a dialog-discursive interview form based on the interview guide and on the results of the focus group interviews. The reason for choosing dialog-discursive interviews with the help of a semistandardized interview guide is that interviews are conducted openly on the one hand, while on the other hand, targeted exploratory questions can be asked regarding specific topics. A semistandardized interview guide is intended to be of assistance, whereby the order of the questions, as well as their wording, are flexibly adapted to the interview situation [47]. The semistandardized interview guide will be developed based on the research results of the scoping review and NPT [41,42] using the SPSS principle, according to Helfferich [60]. The number of interviews is based on the fact that at least 1 interview will be conducted at each management level, for approximately 14 interviews in total. It should be mentioned that the data collection will be determined by an iterative approach. Participants will be recruited by the first author through written information as well as through participation in staff meetings to get to know each other directly. Participation will be strictly voluntary. The providers and work councils (employee representation in the health care provider) will provide informed consent before the data collection step.

All interviews will be recorded using a voice recorder. The transcriptions will be performed by the first author, and after the transcriptions, the audio files will be deleted. The transcription of the interviews will follow the content-semantic transcription process word-for-word. Statements in dialect will be, if possible, translated word-for-word into standard German [61]. Before transcription, the interviews will be stripped of any identifiers so that the participants remain anonymous. Table 1 below shows the coding rules used as an example.

**Table 1:** Exemplary coding rules transcription.

Health care providers and embedded units		Interview	Code
**Provider 1**			
	Case 1.1: long-term care	Focus group interview 1	P1CL1.1F1
	Case 1.2: long-term care	Single interview 2	P1CL1.2S2
**Provider 2**			
	Case 2.1: home care facility	Single interview 4	P2CH2.1S4

##### Data From the Digital Voice Channel

Relevant for ADVICE are the response rate, the frequency of topics mentioned, reactions or responses to comments, and, above all, the consistency of the topics mentioned in the interviews. Furthermore, the digital voice channel data ought to corroborate interview statements, for example, indicating infrequent usage of the free comment fields or the absence of any response. It is important to note that this is an iterative process, using different data to get deeper and deeper in understanding the case.

#### Data Analysis

**Figure 2:**
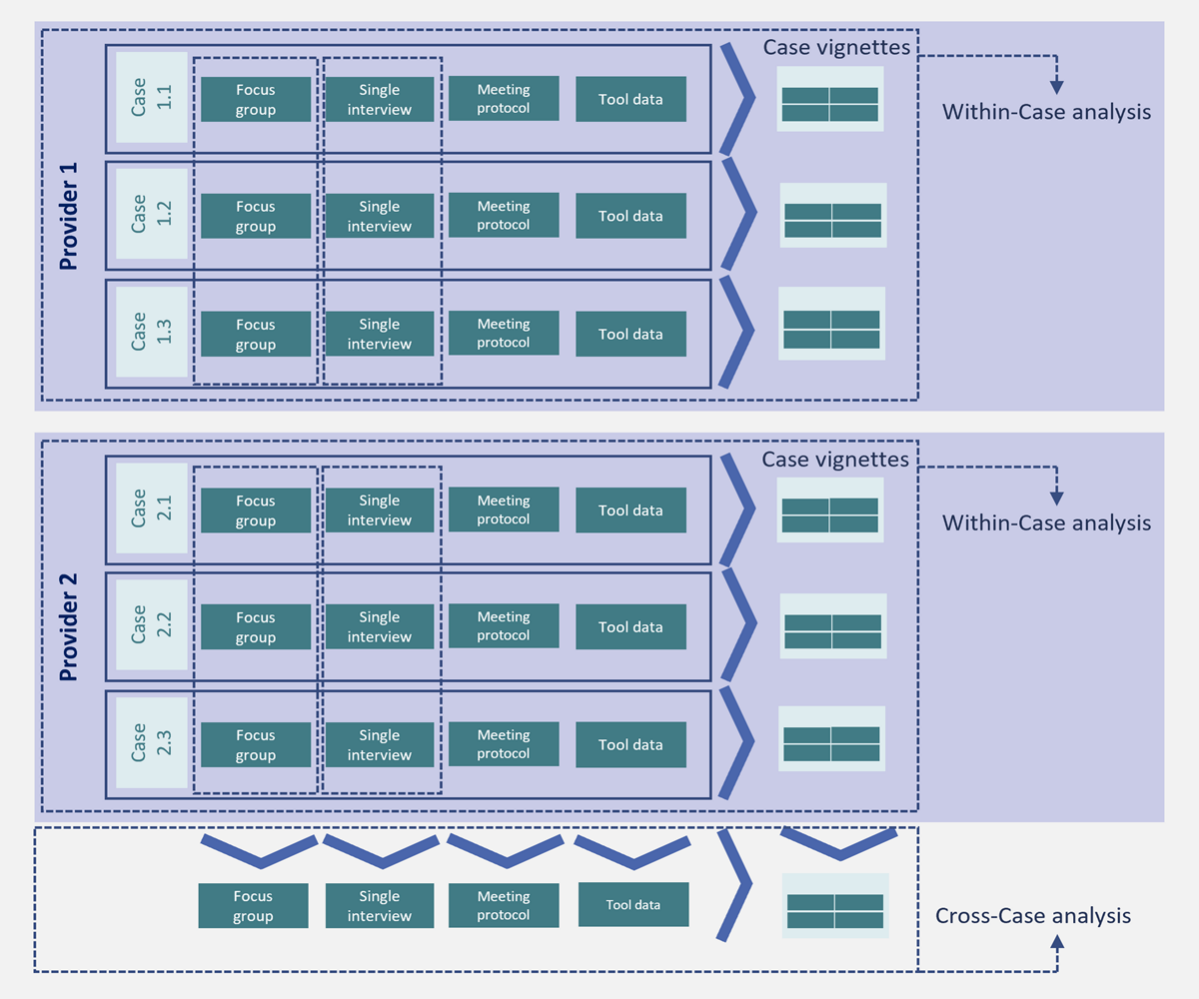
Data analysis.

The principle of data analysis in the case study ADVICE follows the rule of playing with the data and searching for promising patterns, insights, or concepts [44]. Therefore, first, before data analysis, all qualitative and quantitative data will be transferred into MAXQDA Analytics pro software (version 22.2.1; Udo Kuckartz, VERBI software GmbH), where they will be analyzed. Data collection and analysis will be conducted partially in parallel because the case study is an iterative process. The data analysis will be carried out in 2 stages based on the analysis of the individual cases within-case analysis and a subsequent cross-case analysis**,** as shown in Figure 2.

First, in the *within-case analysis*, the individual cases will be analyzed as independent units, and patterns and peculiarities will be identified. The aim is to obtain an in-depth understanding of the specificities of each case. Therefore, all data from every embedded unit will be comprehensively analyzed. For this step, every unit of analysis (focus group interviews, single interviews, protocols, and data from the digital voice channel) will be repeatedly read and reread by the first author. In this iterative process, first, all focus group interviews will be analyzed in light of these results, and the first author will pursue further data collection within the single interviews. Before the interviews are conducted, the interview guide will be updated based on the results from the focus group interviews. Then, all single interviews from every embedded unit will be interpreted. Afterward, a case description will be written for each case with the results of the interviews (plus data from the digital voice channel). This within-analysis is oriented toward qualitative content analysis with regard to Schreier [48].

Based on the research questions, the first author will derive the categories based on the body of knowledge and then further add to or expand on the inductive codes. To be specific, after the first author has determined the deductive category system based on the theoretical framework, all coding units and context units will be determined in consultation with the research team (ie, sample coding). The category system will then be additionally extended by inductive codes. For example, further categories will be formed that are not represented by the coding guide but are relevant to the understanding of the case. Inductive coding will be performed by the first author and selectively verified with other researchers. In summary, a qualitative content analysis with a theoretical deductive framework will be conducted, that is, a deductive-inductive content analysis [48].

In the subsequent second stage of the cross-case analysis, the analyzed data from the embedded units will be compared with each other in a comparative analysis, and possible distortions caused by individual cases will be identified. The results of the within-analysis will provide content for the case vignettes and serve as a basis for the cross-analysis [44]. In this analysis step, the stacking comparable strategy will be used. This strategy includes case-oriented and variable-oriented cross-case analysis strategies. Each case is analyzed with a standard set of variables (based on the results of the within-analysis). After each case is understood in itself (the results will be the case vignettes), a matrix will be used to analyze each case in depth. From this, patterns and differences will be deduced. The case vignettes will include a case study report title, case description summary based on the within-case analysis, analyst view, context information, issues, and uniqueness, among others [45,58]. The goal is to understand how the use of the digital voice channel addresses employee voice not only in every embedded single case but also on a meta-level in long-term care. The presentation of the results will follow the format (graphic, matrix, or networks) proposed by Miles et al [58].

### Ethical Considerations

All participants will receive overall information about the study and be able to agree to participate and to fill out the informed consent form before the interview. Participation will only take place if the written consent form has been signed by the interviewee. The data will be processed in accordance with the data protection legislation in force, pursuant to the GDPR. The personal data collected (age, sex, professional activity, etc) are processed exclusively in an anonymous form and saved separately from the audio data (such as statements made in interviews).

Ethics approval for the empirical parts of the project was given by the ethics committee of Witten/Herdecke University (S230/2022). The ethics vote was submitted under an expedited procedure in accordance with the rules of procedure of the Ethics Committee of the University of Witten/Herdecke, as it does not raise any difficult ethical or legal issues. Furthermore, no review was requested.

## Results

The results will provide insight into how digital voice channels can be used in long-term care to address employee voice. We expect to find how the digital voice channel can empower nurses to speak up and consequently create a better work environment.

The recruitment of the cases from the health care providers took place in consultation with the management and human resources managers. The decision was based, particularly in health care provider 2, on which facilities are already working with the digital voice channel. The recruitment for the focus group interviews and the single interviews began in June 2023.

Data collection began in August 2023, and from a current perspective, the first results are expected in summer 2024.

## Discussion

### Summary

To retain nurses in a health care organization, it is essential that they have a voice and that their views are heard. Reports from nurses working in the Austrian long-term care sector suggest that working conditions need to be addressed [2,3]. The international PTE-LTC program addresses these challenges. Specifically, this program focuses on empowering nurses, giving them a voice to actively participate in improving outcomes for residents [7]. Employee voice is 1 way of involving employees. It means that employees are able to influence work-related decisions through their feedback [12-15].

The current literature shows that employee voice has a positive impact on reducing burnout, promoting job satisfaction [16], and managing change [11,17]. It is important to note that most of the available data on employee voice in health care focuses on 1 aspect of employee voice, namely speaking up regarding patient safety and managing errors [34-36]. This leads to an unclear understanding of the phenomenon of employee voice in health care [36]. Lainidi et al [36] argue that individual approaches and evaluations are needed to gain a deeper understanding of employee voice, especially in different settings. Recent developments in digital and communication technology have opened new opportunities to address employee voice, for example, as a company mirror or digital voice channel [18-20]. Analyses show that a digital voice channel can have a positive impact on employee identification and commitment to the organization [16]. However, it depends on the organizational culture [16,23,24]. In summary, the research gap is the understanding of employee voice in a specific setting, namely long-term care, and the analysis of the use of a digital voice channel. The NPL [41,42] is used in ADVICE to better understand how the social context influences how the digital voice channel gets used to address employee voice.

We assume that the results will provide insight into how digital voice channels can be used in long-term care to address employee voice. We expect to find how the digital voice channel can empower nurses to speak up and, consequently, create a better work environment. Furthermore, we aim to understand how managers deal with the data from the digital voice channel, whether they use the data, and how they respond to trends and comments in the tool. In summary, the results may help to understand how employee voice behavior is affected by the digital voice channel and to identify what opportunities arise for the employees and for the organization through a digital voice channel.

### Strengths and Limitations

We are aware of the different settings, and we try to develop a case understanding of individual cases through our within-case analysis and gain a deeper understanding of employee voice in health care providers by analyzing their similarities and differences. The authors therefore also refer to the NORDCARE report [3], which includes both residential long-term care and mobile care under the term long-term care.

The strength of the method proposed herein lies in the fact that the case study approach addresses the individual experiences of every employee and is oriented to the social processes in the daily routine to understand the use of a new approach to communication (digital voice channel) [44]. These are precisely the aspects that are addressed in the calls for future research on the use of new digital technologies [20,38,39] and employee voice [36]. Potential challenges can be encouraging employees to participate in interviews since these will take up working time, which is very limited due to staff shortages. This challenge can be addressed by attempting to allow the interviews to be completed during duty hours during the recruitment process in consultation with the health care providers. It must be mentioned that the generalizability of case studies is limited based on the high level of specificity and the small number of cases [62]. Furthermore, there is a possibility that, due to the way in which the scope of the data integration of the qualitative and quantitative data will evolve over the course of the work, the case study will also become a mixed methods case study. This may result from the fact that more attention per individual case and subcase is needed in the data integration process that will occur between the qualitative and quantitative research steps [45]. Both interview bias and selection bias must be considered. Interviewer bias is addressed through constant reflection by the research team. The selection of the health care providers is based on the fact that they are the only ones in Austria who use this digital voice channel. The choice of cases is made by the health care provider themselves. Finally, to avoid conflicts of interest, it must be mentioned that the case selection process is undertaken by the company that provides the digital voice channels. It should be noted that the company is not involved in the research process, and moreover, the name of the company is not explicitly mentioned to avoid conflicts of interest.

### Importance of This Study

ADVICE also attempts to address the recommendations of Lainidi et al [36] to consider organizational and employee perspectives to better understand the concepts of voice and silence in a specific context (long-term care). Of course, individual case specifics are considered in the case descriptions, but the main focus is on the digital voice channel used in residential long-term care and home care facilities. At the organizational level, research can help to improve the attractiveness of the workplace by understanding how to give employees a voice.

## Abbreviations

ADVICE: Understanding Employee Voice Behavior (acronym for the study)
CReDECI 2: Criteria for Reporting and Evaluation of Complex Interventions
MeSH: Medical Subject Headings
NPT: normalization process theory
PICO: population, interest, and context
PRISMA ScR: Preferred Reporting Items for Systematic Reviews and Meta-Analyses Extension for Scoping Reviews
PTE-LTP: Pathway to Excellence in Long Term Care
TIDieR: Template for Intervention Description and Replication

## Data Availability

Data sharing is not applicable as this is a protocol paper where no data sets were generated or analyzed.
